# Assessment of menstrual hygiene management knowledge, practice, and associated factors among girls in Boset District, Ethiopia: a school-based cross-sectional study

**DOI:** 10.1186/s40834-023-00233-z

**Published:** 2023-06-01

**Authors:** Wondimagegn Gebre, Endashaw Mandefro Kidane, Yohannes Mekuria Negussie, Mihiret Shawel Getahun, Nardos Tilahun Bekele, Abenet Menene Gurara

**Affiliations:** 1 Department of Public Health, Rift Valley University, Adama, Ethiopia; 2 Department of Medicine, Adama General Hospital and Medical College, Adama, Ethiopia; 3 Department of Nursing, Adama General Hospital and Medical College, Adama, Ethiopia; 4 Department of Public Health, Adama Hospital Medical College, Adama, Ethiopia; 5 Department of Nursing, Arsi University, Asella, Ethiopia

**Keywords:** Menstruation, Menstrual hygiene practice, Adolescent girls, Bostet district, Ethiopia

## Abstract

**Background:**

Adolescent girls, particularly in lower socioeconomic settings, lack adequate knowledge of menstrual hygiene management and have subpar hygiene habits. Likewise, the factors related to it have not been well addressed, and schoolgirls are facing a range of challenges. To develop effective intervention approaches, a context-specific assessment of menstrual hygiene practices is useful. Thus, this study aimed to assess menstrual hygiene management knowledge, practice, and associated factors among girls in the Boset district, Ethiopia.

**Methods:**

A school-based cross-sectional study was conducted at Boset district secondary schools among 629 randomly selected girls using the multi-stage sampling technique. Data were collected using a self-administered, structured questionnaire. The collected data were entered into Epi-info version 7 and analyzed by Statistical Package for Social Science (SPSS) version 26. Binary logistic regression analysis was used to model the association between dependent and independent variables. An adjusted odds ratio and 95% confidence interval were used to measure the strength of the association, and a p-value < 0.05 was used to declare statistical significance.

**Result:**

Of the total study participants, 51.2% (95% CI: 46.6–55.8%) had an appropriate practice of menstrual hygiene and 57.9% (95% CI: 53.3–62.5%) had good knowledge status on menstrual hygiene management. Being an urban resident (AOR = 1.84, 95% CI: 1.20–2.80), having a mother with a secondary and above educational level (AOR = 3.4, 95% CI: 2.07–5.57), earning regular pocket money (AOR = 2.19, 95% CI: 1.45–3.313), and discussing menstrual issues with parents and friends (AOR = 3.65, 95% CI: 2.327–5.727) were associated with good practice of menstrual hygiene.

**Conclusion:**

In this study, nearly half of the school girls had good practice and knowledge of menstrual hygiene management. Educating mothers and promoting discussion about menstrual hygiene management issues, especially in rural areas, should be priority actions.

## Background

Menstruation is a normal part of the female reproductive cycle and biological maturation that begins at puberty, and menstrual hygiene is basic to the dignity and well-being of women and girls as well as an essential component of the basic hygiene, sanitation, and reproductive health services to which each woman and girl has a right [1, 2]. Menstrual hygiene management (MHM) practice is defined as using clean menstrual management material, cleaning the body as necessary with soap and water, and getting the means to dump used materials [3].

Girls’ menstrual coping techniques differ widely between and within countries, based on an individual’s personal choices, resources available, economic position, local customs, cultural values, and expertise or education [4]. But owing to countless cultural and societal misconceptions about menstruation, most of them lack enough information about ways to manage their menstrual hygiene [5].

Worldwide, 2.3 billion people don’t have basic sanitation services, and only 27% of those living in developing countries have the opportunity to have access to water and soap for hand washing [6]. Nearly half of the schools in low-income nations don’t have sufficient drinking water, sanitation, or hygiene, which are essential for girls to deal with their menstruation [7]. In countries in sub-Saharan Africa, the majority of adolescents do not have access to safe, private, clean bathrooms and facilities for washing at school [8].

Girls need to be able to control menstrual bleeding efficiently if they are to live productive, healthy, and comfortable lives. Poor MHM has numerous negative consequences, such as reproductive organ and urogenital infections. Causing a serious detrimental effect, leading to conditions including pelvic inflammatory disease and dysmenorrhea, as well as infertility. Due to agony and dishonor during menstruation, many schoolgirls suffer from concentration problems, restricted engagement, and a loss of confidence in class, which contribute to school absences or dropouts, poor performance, and reduced self-esteem [9–13]. About 10% of African school-age girls and 17% of Ethiopian girls skip school during menstruation [14, 15]. Further, poor MHM can lead to psychosocial anxiety, fewer prospects for education and employment, and a lower quality of life [10, 13].

Particularly in lower socioeconomic settings, adolescent girls’ knowledge about menstruation is limited, and their hygiene practices are improper. This could have clinical implications in terms of integrating the promotion of menstrual hygiene practices into the health care system, and extensive efforts, such as policy implications, are required to enhance girls’ knowledge and safe hygienic practices regarding menstruation, which begin in adolescence [16–18]. Although safe menstrual hygiene practices can help millions of women experience these complicated and complex problems, developing countries, including Ethiopia, have overlooked an opportunity to address the level of understanding and hygienic practices of menstruation among girls as early as adolescence. Despite the official inclusion of menstrual hygiene under reproductive health, the water, sanitation, and hygiene (WASH), education, and sexual and reproductive health sectors did not devote adequate attention [19, 20].

Because menstrual hygiene management has not been extensively understood and the concerns related to it have not been well addressed, schoolgirls may confront a variety of hurdles. While studies on the issue are done in other parts of the country, no study on the same topic has been done in the study area; the magnitude of the problem is therefore unknown, and context-specific or area-relevant pertinent factors are not explored and context-specific assessment of practice may be beneficial in designing tailored measures of intervention. Thus, this study aimed to assess menstrual hygiene management knowledge, practice, and associated factors among girls in the Boset district, Ethiopia. The findings might be of essential clinical value, assisting in the development of policies and suitable intervention strategies. Also, the results of this study will be used as a baseline for further studies.

## Methods and materials

### Study design, setting, and period

A school-based cross-sectional study was employed in the Boset district, East Shewa Zone, of the Oromia region from March 28, 2022, to April 28, 2022. Boset district is located in the eastern part of Ethiopia, 125 km from Addis Ababa, within the Great East African Rift Valley. The total population of this district is predicted to be 220,362, with 112,385 females. In the district, there are 38 elementary schools, 30 junior high schools, and 6 secondary schools. The total number of students enrolled in secondary schools for the academic year 2021/22 was 3,939, with 1,649 being female.

### Population and eligibility criteria

The source population included all secondary school girls who had reached menarche and were enrolled in regular programs at Boset district secondary schools during the study period. All randomly selected secondary school girls who had reached menarche and were enrolled in the regular academic program at the selected schools during the study period made up the study population. Female students who hadn’t started experiencing menses, those enrolled in evening classes, those who were gravely ill at the time the data were collected, those who had cognitive impairment, and those who were absent were all excluded from the study.

### Sample size determination and sampling procedure

The sample size was determined using the single population proportion formula under the assumptions of a 95% confidence interval, a calculated margin of error of 4.6%, and a 34.7% proportion of good practice of menstrual hygiene among school girls in Holeta town [21]. Accordingly, the calculated sample size for this study was 411, and after considering a design effect of 1.5 and 10% non-response, the final sample size becomes 686.

To select study participants, a multistage sampling technique was utilized. Initially, the secondary schools in the district were stratified as private and governmental. Thus, there were six secondary schools (two private and four governmental). Next, at random, 4 schools (two private and two governmental) were chosen, and then each school was stratified into grades, which were then further stratified into sections. The total number of female students in each section was then determined, and the sample size was allocated proportionally. Eventually, a simple random technique was used to select study participants by employing female student lists as sample frames obtained from respective sections.

### Data collection procedure and quality control

Data were gathered using self-administered structured questionnaires adapted from various relevant literature and tailored for the study’s context [21–26]. The questionnaire includes questions on socio-demographic information, knowledge about menstrual hygiene management, and the practice of menstrual hygiene management. The data were gathered by seven trained midwives, supervised by three public health professionals.

Content validity was assessed in consultation with experts in the discipline of public health. The internal consistency of the tools was evaluated using Cronbach’s alpha, and the results indicated that it was good. The tool’s reliability was also confirmed by pre-testing the questionnaire. This contributes to the questionnaire’s structure, clarity, and consistency. It was pre-tested with 5% (n = 35) of the sample, and any required corrections and modifications were undertaken. Data collectors and supervisors received two days of training on data-gathering procedures and the general objective of the study. The principal investigator examined and cross-checked the questionnaire for completeness, correctness, and consistency, and discussions were held with all data collectors daily.

### Operational definitions

#### Knowledge of menstrual hygiene management

Twelve knowledge questions were used to assess knowledge of menstrual hygiene management. Each correct response received one point, whereas any wrong or I don’t know answer received zero points. The mean value was then computed, and the mean score was assigned as the cutoff point. Participants with scores at or above the mean were considered to have adequate or good knowledge about menstrual hygiene management, but those with scores below the mean were deemed to have poor knowledge.

#### Practice of menstrual hygiene management

The menstrual hygiene management practice score was derived from ten practice-specific questions. Each correct response received one point, whereas any wrong or I don’t know answer received zero points. Then, by adding up the practice scores, the cutoff threshold was determined using the mean score. Those who scored at or above the mean were classified as having good menstrual management practices, while those who scored below the mean were classified as having poor practices.

### Data processing and analysis

The data were checked for completeness, coded, and entered into Epi-Info version 7 before being exported to the Statistical Package for Social Sciences (SPSS) version 26 for statistical analysis. The Shapiro-Wilk test was used to validate the normality assumptions for continuous data. Descriptive statistics were employed to explain the study population using relevant variables. The associations between menstrual hygiene management and independent variables were modeled using binary logistic regression analysis. The statistical assumptions of the model, such as multi-collinearity, normality, linearity, residual independence, and outliers, were examined, and no major violations were found. The standard model-building method was used to fit the model. A p-value of < 0.25 was utilized as a cut-off value in the bivariable logistic regression model to select variables for the multivariable logistic regression model to control the possible effects of confounders. The final model’s goodness of fit was assessed using the Hosmer and Lemeshow test, and the result was significant with a p-value ˃ 0.05. The adjusted odds ratio (AOR) with a 95% confidence interval (CI) was used in the final model to identify factors associated with menstrual hygiene management practice. A p-value of < 0.05 was declared statistically significant at this level.

## Results

### Socio-demographic characteristics

This study included 629 female secondary school students, giving it a response rate of 91.7%. The mean age of participants was 17.48 (SD: ±1.12) years and the mean age at menarche was 12.78 (SD: ±0.98) years. Most 416 (66.1%) of the participants were from rural residences, and 451 (71.7%) of them were from governmental schools. Of the total participants, 576 (91.6%) were single, 482(76.6%) Oromo in ethnicity, and 401(63.8%) Orthodox in religion respectively.

In terms of education, 406 (64.5%) respondents’ mothers and 338 (53.7%) respondents’ fathers had no formal education. 405 (64.4%) of the respondents’ mothers were housewives, while 356 (56.6%) of their fathers were farmers. About 250 (39.7%) of respondents’ families had an average monthly income between 2000 and 3000 Ethiopian birr, and 468 (69.6%) get permanent pocket money from their families ***(***Table 1***).***

**Table 1:** Socio-demographic characteristics of secondary school girls in Boset district, Central Ethiopia, 2022 (n = 629)

Variables	Categories	Frequency	Percent
Age (years)	13–18	350	55.6
	≥ 19	279	44.4
Age at menarche (years)	12–14	502	79.8
	> 14	127	20.2
Residence of students	Urban	213	33.9
	Rural	416	66.1
School type	Governmental	451	71.7
	Private	178	28.3
Grade Level	9th to 10th	528	83.9
	11th to 12th	101	16.1
Marital status	Single	576	91.6
	Married	31	4.9
	Engaged	22	3.5
Ethnicity	Oromo	482	76.6
	Amhara	125	19.9
	Gurage	14	2.2
	Others*	8	1.3
Religion	Orthodox	401	63.8
	Muslim	192	30.5
	Protestant	27	4.3
	Others**	9	1.4
Living arrangements	Both parents	478	76.0
	Mother only	53	8.4
	Father only	22	3.5
	Relatives	43	6.8
	Others ***	33	5.3
Mother’s educational status	No formal education	406	64.5
	Primary education	57	9.1
	Secondary and above	166	26.4
Father’s educational status	No formal education	338	53.7
	Primary education	63	10.0
	Secondary and above	228	36.2
Mother’s occupational status	Housewife	405	64.4
	Merchant	97	15.4
	Government employee	34	5.4
	Private employee	49	7.8
	Daily laborer	44	7.0
Father’s occupational status	Farmer	356	56.6
	Merchant	88	14.0
	Government employee	121	19.2
	Private employee	42	6.7
	Others****	22	3.5
Average monthly income of parents (ETB)	< 2000	36	5.7
	2000–3000	250	39.7
	3001–4000	181	28.8
	> 4000	162	25.8
Parents provide pocket money regularly	Yes	438	69.6
	No	191	30.4

**Notes**: *Wolayita, Tigre, ** Wakefena, Adventist, ***Husband, Alone, ****Daily labor, Non-governmental organization employee, Driver

**Abbreviation**: ETB, Ethiopian birr

### Knowledge and Information about menstrual hygiene management

In this study, 472 (75% of girls) got information about menstruation before reaching menarche: 204 (43.2%) from their mothers, 158 (33.3%) from their schools, and 84 (17.8%) from health professionals.

Of the participants, 496 (78.9%) indicated menstruation is a physiological process. About 312 (49.6%) of them knew that the uterus is the origin of the menstrual blood and 351 of them (55.8%) knew that the cause of menstruation is hormonal. Five hundred thirty-six (85.2%) girls reported that the normal menstrual bleeding duration was 2 to 7 days, while 339 (53.9%) of them believed the normal duration of the menstrual cycle was 20–35 days. About 414 girls (65.8%) knew that menstruation is not a lifelong process, 503 (80.0%) were aware that menstrual blood was unhygienic, and 383 girls 399 (60.9%) knew about the availability of sanitary pads in the market. The majority of girls (66.8%) did not freely discuss menstruation issues with their parents and friends. The reasons for not discussing were shamefulness (312, 74.3%), followed by privacy or secrecy menstruation issue 80, (19%) (Table 2).

**Table 2:** Knowledge and information about menstrual hygiene management among secondary school girls in Boset district, Central Ethiopia, 2022 (n = 629)

Variables	Categories	Frequency	Percent
Heard about menstruation before menarche	Yes	472	75.0
	No	157	25.0
Source of information about menstruation (n = 472)	Mother	204	43.2
	School	157	33.3
	Health professionals	84	17.8
	Others	27	5.7
Menstruation	Is a physiological process	496	78.9
	Is a pathological process	31	4.9
	A curse from God	73	11.6
	I don’t know	29	4.6
Source of blood during menstruation	Uterus	312	49.6
	Vagina	222	35.3
	Urinary Bladder	53	8.4
	Abdomen	22	3.5
	I don’t know	20	3.2
Cause of menstruation	Hormone Action	351	55.8
	Curse of God	199	31.6
	Disease	39	6.2
	I don’t know	40	6.4
Mensuration is a lifelong process	Yes	215	34.2
	No	414	65.8
Normal menstrual bleeding duration	< 2 days	43	6.8
	2–7 days	536	85.2
	> 7 days	30	4.8
	I don’t know	20	3.2
The normal duration of the menstrual cycle	< 20	74	11.8
	20–35 days	339	53.9
	> 35 days	145	23.1
	I don’t know	71	11.3
Know menstrual blood is unhygienic	Yes	503	80.0
	No	126	20.0
Know that there is foul smelling	Yes	479	76.2
	No	150	23.8
Know sanitary pads in the market	Yes	383	60.9
	No	246	39.1
Freely discuss with parents, and friends about menstrual issues	Yes	209	33.2
	No	420	66.8
Reason for not discussion with parents, and friends (n = 420)	Shamefulness	312	74.3
	Privacy or secrecy	80	19
	Not habitual	28	6.7
Overall knowledge status	**Good**	**364**	**57.9**
	**Poor**	**265**	**42.1**

### Practice of menstrual hygiene management

In this study, about 378(60.1%) of girls used absorbent materials during their previous menstrual period, and 504 (80.1%) used commercially made disposable sanitary pads. Among the total, 251 (39.9%) changed their absorbent material more than three times a day during menstruation, 378 (60.1%) washed reusable sanitary pads with soap and water, 350 (55.6%) dried reusable sanitary pads in the sunlight, and 251 (39.9%) of them disposed of pads by wrapping them in the paper. Only 125 (19.9%) of the total participants took daily baths with soap and water during menstruation. All of the participants reported cleaning their external genitalia during menstruation, but only 243 (38.6%) reported using water and soap to clean their external genitalia, and nearly half of them (315, (50.1%) disposed of used menstrual materials in waste bins (Table 3).

**Table 3:** Practice of menstrual hygiene management among secondary school girls in Boset district, Central Ethiopia, 2022 (n = 629)

Practice-related questions	Yes		No	
	**Frequency(N)**	**Percent (%)**	**Frequency(N)**	**Percent (%)**
Used absorbent materials during menstruation	378	60.1	251	39.9
Uses commercially made disposable sanitary pads	504	80.1	125	19.9
Changes absorbent more than three times a day	251	39.9	378	60.1
Wash reusable sanitary pads with soap and water	378	60.1	251	39.9
Dry reusable sanitary pad in the sunlight	350	55.6	279	44.4
Disposes of sanitary pads by wrapping them in paper	251	39.9	378	60.1
Takes bath daily with soap and water during menstruation	125	19.9	504	80.1
Cleans external genitalia during menstruation	629	100	386	61.4
Cleans external genitalia with water and soap	243	38.6	0	0
Disposes of used menstrual materials in waste bins	315	50.1	314	49.9

### Magnitude of practice of menstrual hygiene management

**Fig. 1:**
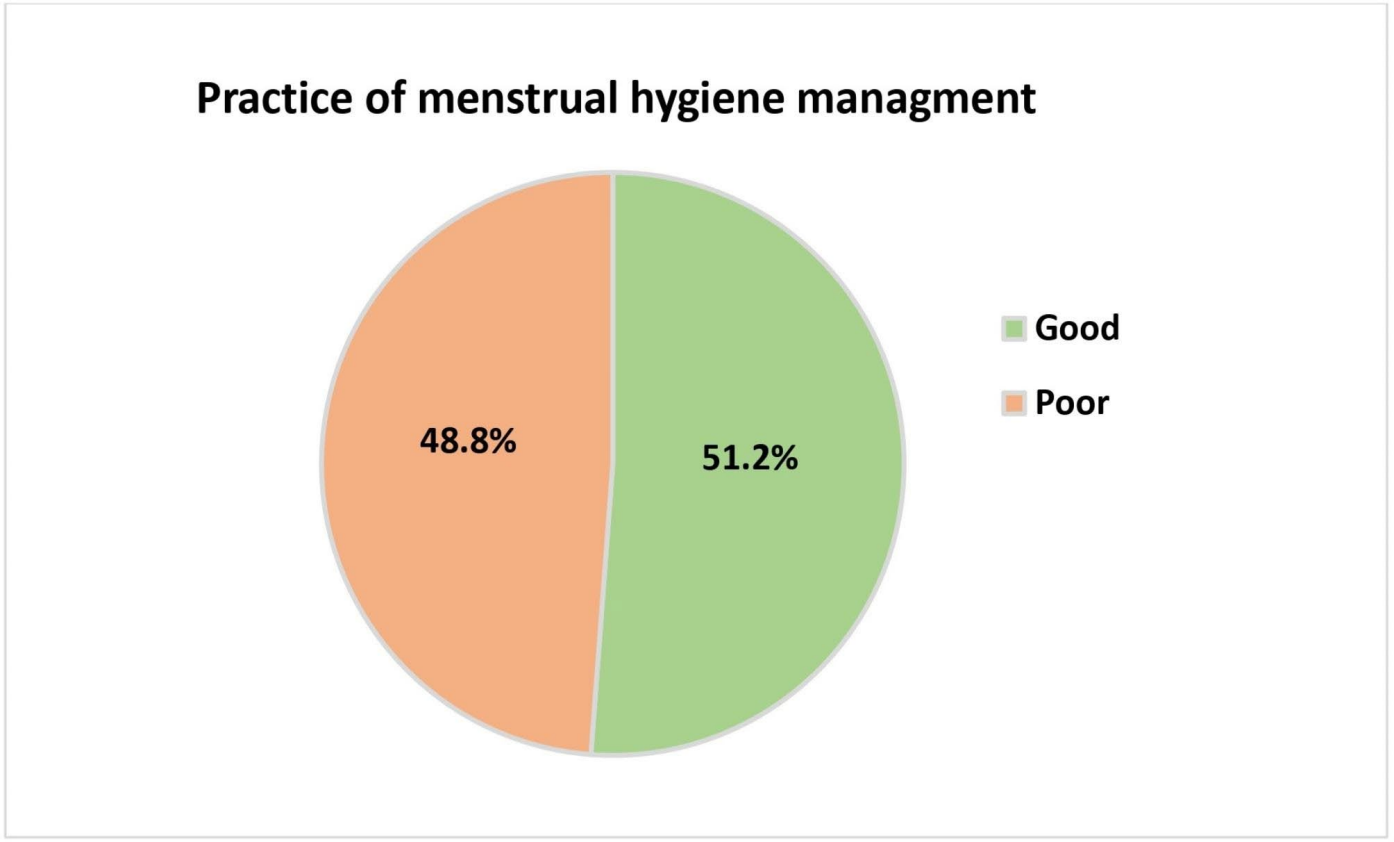
Magnitude of menstrual hygiene management practice among secondary school girls in Boset district, Central Ethiopia, 2022

The overall magnitude of good menstrual hygiene management practice among school girls in the Boset district was 322 (51.2%) with (95% CI: 46.6–55.8%) (Fig. 1).

### Factors associated with the practice of menstrual hygiene management

 To assess the association between the independent variables and the practice of menstrual hygiene management, bivariate, and multivariate analyses were used. At a p-value < 0.25, the factors in the bivariate analysis that exhibited an association with the outcome variable were chosen as candidate variables for multivariable logistic regression analysis.

 In the bivariate analysis, residence area, educational status of the mother, educational status of the father, receiving regular pocket money from parents, heard about menstruation before menarche, freely discussing menstrual issues with parents and friends, and overall knowledge about menstruation hygiene management showed statistically significant association. Following adjustment for potential confounding factors with multivariable binary logistic regression analysis, residence area, educational status of the mother, receiving regular pocket money from parents, and freely discussing menstrual issues with parents and friends persisted as statistically significant factors associated with the practice of menstrual hygiene management at a p-value < 0.05.

 Hence, the odds of having a good practice of menstrual hygiene management were 1.84 times higher among girls who were urban residents compared with those who were rural residents (AOR = 1.84, 95% CI: 1.20–2.80). Girls who had mothers with a secondary and above educational level had 3.4 times greater odds of good practice in menstrual hygiene management compared to those whose mothers had no formal education (AOR = 3.4, 95% CI: 2.07–5.57). Compared to girls with no regular pocket money, those who had regular pocket money had 2.19 times the odds of having good menstrual hygiene management practices (AOR = 2.19, 95% CI: 1.45–3.313). Moreover, girls who freely discussed menstrual issues with their parents and friends had 3.65 times greater odds of practicing good menstrual hygiene management than their counterparts (AOR = 3.65, 95% CI: 2.327–5.727) (Table 4).

**Table 4:** Factors associated with the practice of menstrual hygiene management among secondary school girls in Boset district, Central Ethiopia, 2022

Variables	Category	MHM practice		COR (95% CI)	AOR (95% CI)
		**Good**	**Poor**		
Residence	Urban	131	82	1.88(1.34,2.64) *	1.84(1.20,2.80) **
	Rural	191	225	1	1
Mother’s educational status	Secondary and above	126	40	4.33(2.88,6.5) *	3.4(2.07,5.57) **
	Primary education	25	32	1.07(0.6,1.88)	1.20(0.62,2.301)
	No formal education	171	235	1	1
Father’s educational status	Secondary and above	117	111	0.99(0.71,1.39)	0.99(0.71,1.396)
	Primary education	31	32	0.91(0.53,0.99) *	0.907(0.52,1.57)
	No formal education	174	164	1	1
Parents provide pocket money regularly	Yes	247	191	2.0(1.42,2.83) *	2.19(1.45,3.313) **
	No	75	116	1	1
Heard about menstruation before menarche	Yes	241	231	0.98(0.68,0.999)	1.045(0.70,1.55)
	No	81	76	1	1
Freely discuss with parents, and friends about menstrual issues	Yes	159	50	5.01(3.45,7.28) *	3.65(2.327,5.727) **
	No	163	257	1	1
Knowledge status	Good	203	161	1.55(1.13,2.12) *	1.156(0.68,1.95)
	Poor	119	146	1	1

**Notes** *Significant at p-value < 0.25 in unadjusted logistic regression analysis, ** significant in adjusted logistic regression analysis, 1 = reference.

**Abbreviations**: COR: crude odds ratio; CI: confidence interval; AOR: adjusted odds ratio.

## Discussion

This study aimed to assess menstrual hygiene management knowledge, practice, and associated factors among girls in the Boset district, Ethiopia. The magnitude of good practice of menstrual hygiene management was 51.2%, and 57.9% had good knowledge status on managing menstrual hygiene. Residence, educational status of the mother, receiving regular pocket money from parents, and freely discussing menstrual issues with parents and friends were significant predictors of the practice of menstrual hygiene management.

This study found that good practice of menstrual hygiene management was 51.2% (95% CI: 46.6–55.8%). This finding is in agreement with studies conducted in Dessie (53.9%) [27], Ghana (50.8%) [28], and West Bengal (47.5%) [3]. However, it was lower than studies done in East Hararghe (58.3%) [24], Adama (57.0%) [29], Mekidela (62.4%) [30], and Nepal (67%) [26]. Moreover, the practice of menstrual hygiene management in the current study was higher than in studies done in Bahir Dar (24.5%) [31], Holeta (34.7%) [21], Habru (35.4%) [13], Ambo (46.7%) [32], Gimbi[33], Wegera district (29.8%) [34], Sebeta town(21%) [35], Nekemte (39.9%) [22], Southern Ethiopia (39.7%) [23] and a systematic review and meta-analysis done in sub-Saharan Africa (45%) [36]. This discrepancy may be explained by variations in the socioeconomic features of the study areas, the study population, the study period, and the assessment methods employed to evaluate the management of menstrual hygiene practices.

In this study, 57.9% (95% CI: 53.3–62.5%) of girls had good knowledge status on managing menstrual hygiene. This is comparable with a study conducted in Nekemte (60.9%) [22], but lower than studies conducted in Gimbi (64.8%) [33], Mekidela (64.9%) [30], Holeta (72.5%) [21] and East Hararghe (68.5%) [24]. This finding was higher compared to the findings from Wegera (34.3%) [34], and Southern Ethiopia (31.7%) [23]. The discrepancy might result from differences in sample size, study setting, accessibility, and information supplied by schools and families, as well as communication about menstruation and menstrual hygiene issues within families.

The odds of having a good practice of menstrual hygiene management were 1.84 times higher among girls from urban residents compared with those from rural residents. this finding is supported by studies done in Gimbi[33], Harar[37], Holeta[21], Batu town[38], East Hararghe[24], and Mekidela[30], a systematic review and meta-analysis conducted in sub-Saharan Africa[36] and India[39]. This could be because, in comparison to girls who come from rural regions, girls from urban areas are more exposed to information and services about sexual and reproductive health concerns, such as menstruation issues. In addition, there is a lack of affordability and accessibility to sanitary products, functional latrines, hygiene, and sanitation facilities in rural areas, which puts girls at a distinct disadvantage when it comes to maintaining their menstrual hygiene[19, 40].

Girls who had mothers with a secondary and above educational level had 3.4 times greater odds of good practice in menstrual hygiene management compared to those whose mothers had no formal education. This finding is similar to studies conducted in Nekemte[22], East Hararghe[24], Bahir Dar[31], Gimbi[33], Sebeta town[35], Eastern Ethiopia[41], a systematic review and meta-analysis conducted in sub-Sharan Africa[36], and studies from India and Lebanon[42, 43]. This can be justified by the fact that educated mothers might be more cognizant of menstrual hygiene practices; they may be capable of engaging in a dialogue with their daughters concerning menstruation and providing proper sanitary supplies to ensure that they maintain their menstrual hygiene.

In this study, compared to girls with no regular pocket money, those who had regular pocket money had 2.19 times the odds of having good menstrual hygiene management practices. This finding is consistent with a systematic review and meta-analysis in Ethiopia[44], and a study in South India[25]. A study done in Eastern Ethiopia also found that the likelihood of poor practice of menstrual hygiene management was 51% less for girls with regular pocket money compared with their counterparts[41]. The justification could be that girls who receive monetary support from their parents can readily purchase sanitary materials for menstruation hygiene which may result in girls practicing good menstrual hygiene. Moreover, pocket money is identified as a key intervention to meet menstrual hygiene requirements, and also access to quality hygiene items to encourage proper menstrual hygiene[45].

Discussions with parents, and friends about menstrual issue was another significant factor associated with menstrual hygiene management practices. Girls who freely discussed menstrual issues with their parents and friends had 3.65 times greater odds of practicing good menstrual hygiene management than their counterparts. this is in line with studies undertaken in Gimbi[33], Dessie[27] and India[46]. This could be because talking about menstruation with parents and friends can help in learning more about it, reduce psychological stress, and boosts confidence. which, in turn, increases the practice of good menstrual hygiene management.

### Limitations of the study

Because of the cross-sectional study design utilized in this study, it is impossible to identify temporal relationships, and hard to confirm the causal-effect link between the dependent and predictor variables. The adequacy of the school’s infrastructure to practice good menstrual hygiene and the school programs that may impact the student’s knowledge of menstrual hygiene management weren’t addressed extensively. Also, there might be recall and social desirability biases due to the sensitivity of menstrual issues.

## Conclusion

Nearly half of the schoolgirls in the Boset district had good practice and knowledge of menstrual hygiene management. Residence, the mother’s educational level, having regular pocket money from parents, and being able to openly discuss menstrual issues with friends and family were independent predictors of the practice of menstrual hygiene management.

Strengthening menstrual hygiene awareness and advocacy activities through school media and special education initiatives in schools is necessary to promote menstrual hygiene understanding and practice. It’s also crucial to encourage parent-adolescent girls’ discussions about menstruation. Likewise, healthcare facilities, the media, and other stakeholders should develop suitable health education and awareness-raising programs for girls and the general public on menstrual hygiene management practices, as well as encourage discussion of menstrual hygiene management issues, particularly in rural areas. Maternal education should also be promoted as part of a government-level public health strategy to prevent associated health issues and strengthen the safe practice of menstrual hygiene management.

## Data Availability

All data and materials are available from the corresponding author without undue reservation.

## Abbreviations

AOR: Adjusted Odds Ratio
COR: Crude Odds Ratio
CI: Confidence Interval
MHM: Menstrual Hygiene Management
SPSS: Statistical Package for Social Sciences
